# COVID-19 preventive social-behavioural practices and exposure to SARS-CoV-2 among residents in the city of Yaounde: Lessons from the early phase of the pandemic in Cameroon

**DOI:** 10.1371/journal.pgph.0002331

**Published:** 2023-08-30

**Authors:** Joseph Fokam, Alex Durand Nka, Jeremiah Efakika Gabisa, Kene Nwosu, Franck Wanda, Lucien Mama, Aude Christelle Ka’e, Yagai Bouba, Ezechiel Ngoufack Jagni Semengue, Michel Carlos Tommo Tchouaket, Désiré Takou, Aurelie minelle Kengni Ngueko, Willy Pabo, Samuel Martin Sosso, Olivia Keiser, Carlo-Federico Perno, Vittorio Colizzi, Edie-Gregory Halle Ekane, John Otshudiema Otokoye, Alexis Ndjolo, Laura Ciaffi

**Affiliations:** 1 Chantal BIYA International Reference Centre for Research on HIV/AIDS Prevention and Management (CIRCB), Yaounde, Cameroon; 2 Faculty of Health Sciences, University of Buea, Buea, Cameroon; 3 National Public Health Emergencies Operations Coordination Centre, Yaounde, Cameroon; 4 Institute of Global Health, University of Geneva, Geneva, Switzerland; 5 Centre International de Recherches, d’Enseignements, et de Soins (CIRES), Akonolinga, Cameroon; 6 Health District of Cite Verte, Regional Delegation of Public Health, Yaounde, Cameroon; 7 National AIDS Control Committee, Yaounde, Cameroon; 8 Bambino Gesu Pediatric Hospital, Rome, Italy; 9 Faculty of Science and Technology, Evangelic University of Cameroon, Bandjoun, Cameroon; 10 World Health Organization (WHO), Cameroon Country Office, Yaounde, Cameroon; Emory University School of Medicine, UNITED STATES

## Abstract

Non-pharmaceutical interventions remain key in mitigating the spread of SARS-CoV-2. We sought to assess COVID-19 preventive, social-behavioural practices, and SARS-CoV-2 exposure through IgG rapid tests. This was a cross-sectional survey among 971 respondents residing in 180 households within the “*Cite Verte*” health district of Yaounde-Cameroon, from October-November 2020. Using a structured questionnaire, data on SARS-CoV-2 preventive and social behavioural practices were collected, while exposure to SARS-CoV-2 was determined by IgG profiling. p<0.05 was considered statistically significant. Overall, 971 participants were enrolled, among whom 56.5% were females. The age group 15–29 (33.5%) and those with a secondary level of education (44.7%) were most represented. Regarding preventive/social behavioural practices, the least respected measure was "stopped work", 49.1%, while the most respected was "Respect of hygiene rules", 93.8%. Women obeyed preventive measures more than men, with 87.6% vs 81.0% adhering to the lockdown, (p = 0.005) and 95.5% vs 91.7% to hygiene rules (p = 0.017). The age range 45–64 years was the least adherent to the lockdown rule, with 75.2% (38/153), p<0.0001. Only 24.7% (73/295) and 6.1% (59/295) of the symptomatic individuals reported having sought medical consultation and Covid-19 testing respectively. In addition, up to 69.8% (555/795) felt healthcare facilities were high-risk sites for getting infected, p = 0.002. Exposure to SARS-CoV-2 by IgG positivity was 31.1% (302/971), with men recording a higher proportion of viral exposure, 51.0% (154/302), p = 0.021. After adjusting for gender, age, education, and occupation; salaried worker (p = 0.029; OR: 0.29), and trading (p = 0.001; OR: 0.23) least complied with lockdown rule. In this community of Cameroonian residents highly exposed to COVID-19, many perceived healthcare facilities as high-risk zones for SARS-CoV-2 infection and consequently did not seek medical interventions. Thus, in the context of such a pandemic, advocacy on risk communication and community engagement for health-seeking attitudes should preferentially target men and those afraid of pandemics.

## Introduction

The SARS-CoV-2 was first reported in Wuhan-China in late December 2019: it spread across the world swiftly, affecting thousands of persons within a short while. Consequently, on January 30, 2020, the WHO declared the outbreak a public health emergency of international concern and later characterised it as a pandemic on March 11, 2020 [1].

COVID-19 has a zoonotic origin [2] and transmission from person to person occurs mainly via respiratory droplets, either by being inhaled or deposited on mucosal surfaces, including aerosols produced when coughing and speaking. The COVID-19 incubation period, epidemiologic characteristics, and basic reproductive number are key factors influencing the spread of the disease. Its clinical features mimic those of other illnesses and vary from no symptoms (asymptomatic), to mild, moderate, or severe or fatal symptomatic illness. The most common symptoms include fever, cough, and myalgia. Other minor symptoms are sore throat, headache, chills, nausea or vomiting, diarrhoea, ageusia, and conjunctival congestion. WHO in early 2020 reported that approximately 80% of patients infected with COVID-19 showed mild symptoms or were asymptomatic, and eventually recovered without any medical intervention, whereas 15% of infected persons presented with severe illness, including shortness of breath, septic shock, and multiple organ failure [3].

Non-pharmaceutical COVID-19 mitigation measures like the regular wearing of face masks, practicing social distancing, and isolating suspected and confirmed cases, as well as community containment, washing of hands with soap under running water, and observation of sneezing and coughing etiquette, have played a key role in mitigating the spread of the epidemic [4, 5].

The role of information, education, and communication in promoting the practices of these non-pharmaceutical preventive measures cannot be underscored. The WHO action plan for COVID-19 preparedness and response highlights risk communication and community engagement strategies aimed at protecting individuals, families and curb the virus transmission [6]. China has implemented all these non-pharmaceutical measures, with unprecedented efforts, and successfully curbed the spread of the virus across the country in a relatively short period [7, 8].

The assertion that black Africans are less prone to severe manifestations [9–11] together with political distrust precipitated non-adherence to public health interventions intended to help curb the viral spread [12, 13]. A study conducted in Ivory Coast noted that many people considered preventive measures as “antisocial” [14]. Many in Cameroon perhaps shared the same assertion and did not adhere to protective recommendations strictly; consequently, the infection rate in the country rose from 3 cases as of March 09, 2020, to a cumulative 21543 cases as of October 12, 2020 (with a cumulative death rate of about 2.0%, 425/21543) [15].

In view of the considerable increase in the number of confirmed positive cases, despite the various preventive measures taken by the Cameroon health system, it is important to determine the various factors associated with COVID-19 transmission. This study therefore aimed to assess SARS-CoV-2 preventive social behavioural practices and SARS-CoV-2 IgG among residents in a health district in the city of Yaounde, Cameroon. Specifically, we aimed to identify gaps in practices that may facilitate the silent transmission of the virus through SARS-CoV-2 IgG, as well as loopholes in communication strategies that are crucial in curbing COVID-19 in our context.

## Methods

### Ethics statement

The study protocol obtained the ethical clearance (N° 2020/09/1292/CE/CNERSH/SP) from the national ethics committee and the administrative authorization of the Ministry of Public Health of Cameroon (N°D30-845/L/MINSANTE/SG/DROS). All members of the survey team were trained in health research ethics and good clinical practice. Written informed consent was obtained from the parent/guardian of each participant under 18 years of age.

### Study design, sampling, and setting

We conducted a descriptive, cross-sectional survey within “Cité Verte”, a health district of Yaounde, Cameroon. “Cité Verte” has a cosmopolitan population, including people with diverse educational backgrounds and from different walks of life. The Yaounde Central hospital situated in the cite Cite verte District, is one of the prominent testing and treatment sites within the country. Using a single-stage cluster sampling design, we effectively sampled a total of 180 households within this target health district. Households were randomly selected from a pre-processed set of residential buildings based on OpenStreetMap data [16].

### Inclusion criteria and definitions

Only households whose family heads voluntarily agreed to sign the study’s consent form were included. Within participating households, all individuals between 05 and 80 years of age were included if they had been present in the household for at least 14 days before the survey. Those ≥ 21 years signed a consent form, while those < 21 years were only enrolled upon formal approval by their respective parent(s) or guardian(s); all those < 21 years enrolled were those who assented to willingly participate.

### Data collection tools and procedure

Data collection took place between October 14 and November 26, 2020, using KoboCollect (version 1.29.3–1). In the field, each sampled household was visited by study investigators, who either interviewed residents during the first meeting or arranged an appointment for a future interview if household members were not all present.

### The following information was collected

Demographic information (gender, age), COVID-19 perception (fear of viral contagion and perceived risk of infection) including testing and healthcare perceptions (hospital perception), past and/or present clinical symptoms during the interview sessions.

*COVID-19 compatible symptoms* are symptoms that are similar to those experienced by individuals infected with SARS-CoV-2 but may also be caused by other conditions. They include fever, cough, coryza, headache, myalgia, dyspnoea, sore throat, fatigue, nausea/vomiting.

*COVID-19 symptoms* are symptoms that are more specific to those caused by SARS-CoV-2. They included an acute onset of fever and cough plus at least three of the following symptoms: coryza, headache, myalgia, dyspnoea (breathing difficulty), sore throat, fatigue, nausea/vomiting, and anosmia (loss of sense of smell)/ageusia (loss of sense of taste).

*COVID-19 moderate symptoms included*: Fever, fatigue, and cough, with oxygen saturation ≥94% [17]. Less common symptoms include coryza, headache, myalgia, sore throat, fatigue, nausea/vomiting, anosmia/ageusia.

*COVID-19 severe symptoms mainly included*: Dyspnoea, chest pain, and delirium, with oxygen saturation <94% [17].

*Respect of hygiene rules included*: The regular wearing of face masks, proper sneezing/coughing etiquettes, and proper and regular hand washing.

*Social distancing rules included* no handshakes, no hugging, no intimate contact, no unnecessary outings, and the maintenance of at least 1–1.5 m physical distancing when among people.

*The Lockdown rule* applied to the observance of general curfews which included suspension gathering of about 50 persons or more, general curfew from 6:pm to 6:am in major towns and cities within the country.

*Barrier measures* involved all preventive measures in general and includes respect of hygiene, social distancing, and lockdown rules.

### Testing procedure

The Abbott Panbio COVID-19 IgG/IgM Rapid (Abbott Diagnostics Inc, USA) test device was used to screen for blood SARS-CoV-2 IgG/IgM. This is an immunochromatographic, lateral flow test for the qualitative detection of IgG antibodies to the nucleocapsid (N) protein of SARS-CoV-2. The test has a manufacturer-estimated sensitivity and specificity of 95.8% and 94%, respectively.

### Data processing and statistical analysis

Data were entered into Microsoft Excel 2013 and analyzed using the statistical software SPSS version 21. Bivariate analysis was done using Fischer’s exact and Chi-square test to determine SARS-CoV-2 preventive social behavioural practices and SARS-CoV-2 IgG positivity associated factors. All p-values < 0.05 were considered statistically significant.

## Results

### Clinical characteristics of the study population

One hundred and eighty (180) households participated in this study, resulting in a final sample of 971 respondents. The median age was 26 years (IQR: 14–38), and 56.5% (549/971) of them were female. The majority 44.7% (434/971) had a secondary level of education. Students and informal workers were more represented, 41.4% (402/971) and 20.3% (197/971) respectively (Table 1).

**Table 1:** Clinical characteristics of the study population.

Variables	Respondent’s	Percentage (%)
**Gender**		
Male	422	43.5
Female	549	56.5
**Age (years)**		
5–14.	241	24.8
15–29	325	33.5
30–44	212	21.8
45–64	153	15.8
≥65	40	4.1
**Level of Education**		
No formal Education	57	5.9
Primary	318	32.7
Secondary	434	44.7
Tertiary	145	14.9
Undergraduate/Postgraduate	17	1.8
**Occuapation**		
Housewife/maiden	74	7.6
Student	402	41.4
Salaried worker	54	5.6
Informal worker	197	20.3
Trading	116	11.9
Unemployed	68	7.0
Retiree	32	3.3
Farming	6	0.6
Others	22	2.3
**Symptoms**		
***COVID-19 compatible symptoms***		
YES	295	30.4
NO	676	69.6
***COVID-19 symptoms***		
YES	115	39.0
NO	180	61.0
***Symptom severity***		
Severe	45	15.3
Moderate	250	84.7

Based on symptoms, among the survey respondents retained, 30.4% (295/971) and 39.0% (115/295) presented COVID-19 compatible and COVID-19 symptoms respectively. In terms of symptom severity, 15.3% (45/295) of these symptomatic respondents reported having experienced severe clinical manifestations, while 84.7% (250/295) experienced moderate manifestations (Table 1).

### SARS-CoV-2 preventive and social-behavioural practices

**Fig 1:**
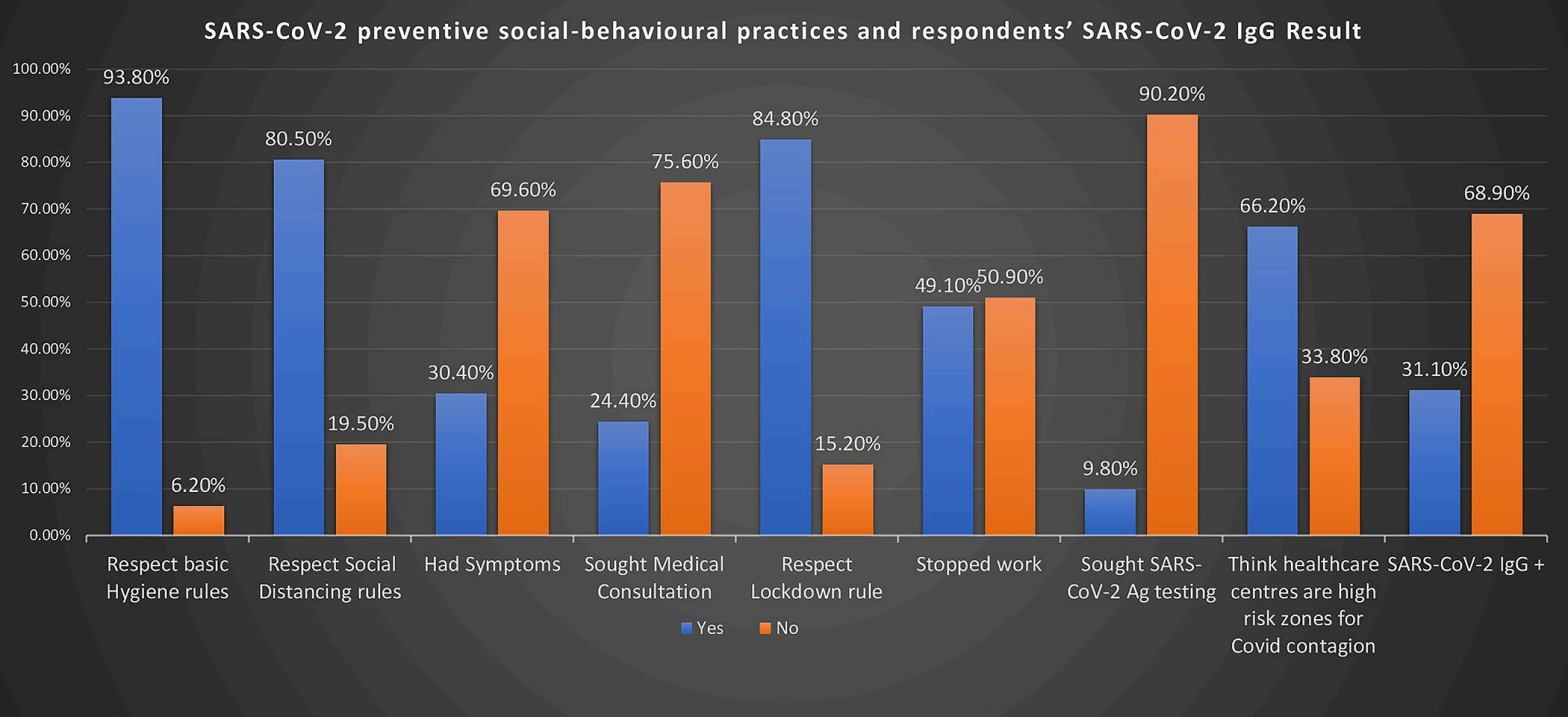
SARS-CoV-2 preventive social-behavioural practices and respondents’ SARS-CoV-2 IgG result. COVID-19 compatible symptoms include at least one or more of fever, cough, coryza, headache, myalgia, dyspnea, sore throat, fatigue, nausea/vomiting, anosmia/ageusia. COVID-19 symptoms include an acute onset of fever and cough plus at least three of the following symptoms coryza, headache, myalgia, dyspnea, sore throat, fatigue, nausea/vomiting, anosmia/ageusia. Respect of hygiene rules include the regular wearing of face masks, proper sneezing etiquettes, and proper and regular handwashing. Respect social distancing rules include no handshakes, no hugging, no intimate contacts, no unnecessary outings, and the maintenance of at least 1–1.5m social distancing space when in the midst of people. Lockdown rule applies to the observance of general curfews. Stopped work included all those who stopped going to work in fulfilment of protection protocols.

Overall, the most commonly observed preventive measure was adherence to hygiene rules, 93.8% (911/971), followed by adherence to the lockdown 84.8% (823/971), and social distancing rules, 80.5% (782/971). Four hundred and ninety-four 50.9% (494/971) continued to work despite the lockdown rule. The general perception of healthcare facilities was poor, with up to 66.2% (643/971) referring to healthcare centers as high-risk zones for SARS-CoV-2 contagion. Only 24.4% (72/295) and 9.8% (29/295) of symptomatic respondents reportedly sought medical consultation and SARS-CoV-2-Ag testing respectively (Fig 1).

The majority, 81.9% (795/971) of respondents were afraid of getting the virus. Regarding the perceived risk of getting infected, 61.0% (592/971) felt they have the same risk of infection as any other person, while 14.1% (137/971) and 24.9% (242/971) felt they were more and less likely to get infected, respectively.

### SARS-CoV-2 preventive and social-behavioural practices according to demographic factors

Gender comparison showed higher adherence among women to preventive recommendations; 87.6% (481/549) versus 81.0% (342/422), p = 0.005 for adherence to lockdown (Table 2), 83.2% (457/549) versus 77.0% (325/422), p = 0.016 for social distancing (Table 3), and 95.5% (524/549) versus 91.7% (387/422), p = 0.017 for hygiene (Table 4). The same applied to SARS-CoV-2 testing where women recorded 6.4% (35/549) testing compared to 5.7% (24/422) among men, p = 0.736 (Table 5). More men than women sought medical care (7.8% (33/422) versus 7.3% (40/549), p = 0.761), (Table 6).

**Table 2:** Respect of lockdown rule and associated factors.

Variables	Respondent (N)	Respect lockdown rule		p-value
		**YES**	**NO**	
**Gender**				
Male	422 (43.5)	342 (81.0)	**80 (19.0)**	*0*.005*
Female	549 (56.5)	**481 (87.6)**	**68 (12.4)**	
**Age (years)**				
5–14.	241 (24.8)	228 (94.6)	13 (5.4)	
15–29	325 (33.5)	269 (82.8)	56 (17.2)	
30–44	212 (21.8)	172 (81.1)	40 (18.9)	<0.0001*
45–64	153 (15.8)	115 (75.2)	38 (24.8)	
≥65	40 (4.1)	40 (100.0)	0 (0.0)	
**Level of Education**				
No formal Education	57 (5.9)	46 (80.7)	11 (19.3)	
Primary	318 (32.7)	284 (89.3)	34 (10.7)	
Secondary	434 (44.7)	365 (84.1)	69 (15.9)	0.026*
Tertiary	145 (14.9)	114 (78.6)	31 (21.4)	
Undergraduate/Postgraduate	17 (1.8)	14 (82.4)	3 (17.6)	
**Occupation**				
Housewife/maiden	74 (7.6)	68 (91.9)	6 (8.1)	
Student	402 (41.4)	367 (91.3)	35 (8.7)	
Salaried worker	54 (5.6)	38 (70.4)	16 (29.6)	
Informal worker	197 (20.3)	159 (80.7)	38 (19.3)	<0.0001*
Trading	116 (11.9)	78 (67.2)	38 (32.8)	
Unemployed	68 (7.0)	60 (88.2)	8 (11.8)	
Retiree	32 (3.3)	28 (87.5)	4 (12.5)	
Farming	6 (0.6)	6 (100.0)	0 (0.0)	
Others	22 (2.3)	19 (86.4)	3 (13.6)	
**Symptoms**				
***COVID-19 compatible symptoms***				
YES	295 (30.4)	233(79.0)	62(21.0)	
NO	676 (69.6)	590 (87.3)	86 (12.7)	*0*.001*
***COVID-19 symptoms***				
YES	115 (39.0)	87 (75.7)	28 (24.3)	
NO	180 (61.0)	145 (80.6)	35 (19.4)	0.272
***Symptom severity***				
Severe	45 (15.3)	36 (80.0)	9 (20.0)	
Moderate	250 (84.7)	196 (78.4)	54 (21.6)	0.608
**Fear of contagion and perceived risk of infection**				
***Fear of viral contagion***				
YES	795 (81.9)	**686 (86.3)**	**109 (13.7)**	
NO	176 (18.1)	**137 (77.8)**	**39 (22.2)**	*0*.005*
***Perceived risk of infection***				
same as other persons	592 (61.0)	**511 (86.3)**	**81 (13.7)**	
More than other persons	137 (14.1)	**99 (72.3)**	**38 (27.7)**	*<0*.0001*
Less than other persons	242 (24.9)	**213 (88.0)**	**29 (12.0)**	

**Table 3:** Respect of social distancing and associated factors.

Variables	Respondent (N)	Respect social distancing		p-value
		**YES**	**NO**	
**Gender**				
Male	422 (43.5)	325 (77.0)	**97 (23.0)**	*0*.016*
Female	549 (56.5)	**457 (83.2)**	**92 (16.8)**	
**Age (years)**				
5–14.	241 (24.8)	187 (77.6)	54 (22.4)	
15–29	325 (33.5)	258 (79.4)	67 (20.6)	
30–44	212 (21.8)	172 (81.1)	40 (18.9)	0.221
45–64	153 (15.8)	126 (82.4)	27 (17.6)	
≥65	40 (4.1)	40 (100.0)	0 (0.0)	
**Level of Education**				
No formal Education	57 (5.9)	51 (89.5)	6 (10.5)	
Primary	318 (32.7)	256 (80.5)	62 (19.5)	
Secondary	434 (44.7)	342 (78.8)	92 (21.2)	0.444
Tertiary	145 (14.9)	119 (82.1)	28 (17.9)	
Undergraduate/Postgraduate	17 (1.8)	14 (82.4)	3 (17.6)	
**Occupation**				
Housewife/maiden	74 (7.6)	66 (89.2)	8 (10.8)	
Student	402 (41.4)	312 (77.6)	90 (22.4)	
Salaried worker	54 (5.6)	44 (81.5)	10 (18.5)	
Informal worker	197 (20.3)	165 (83.8)	32 (16.2)	0.131
Trading	116 (11.9)	87 (75.0)	29 (25.0)	
Unemployed	68 (7.0)	57 (83.8)	11 (16.2)	
Retiree	32 (3.3)	26 (81.2)	6 (18.8)	
Farming	6 (0.6)	6 (100.0)	0 (0.0)	
Others	22 (2.3)	19 (86.4)	3 (13.6)	
**Symptoms**				
***COVID-19 compatible symptoms***				
YES	295 (30.4)	216 (73.2)	79 (26.8)	
NO	676 (69.6)	566 (83.7)	110 (16.3)	*<0*.0001*
***COVID-19 symptoms***				
YES	115 (39.0)	78 (67.8)	37 (32.2)	
NO	180 (61.0)	137(76.1)	43 (23.9)	0.100
***Symptom severity***				
Severe	45 (15.3)	36 (80.0)	9 (20.0)	
Moderate	250 (84.7)	179(71.6)	71 (28.4)	0.159
**Fear of contagion and perceived risk of infection**				
***Fear of viral contagion***				
YES	795 (81.9)	661 (83.1)	134 (16.9)	
NO	176 (18.1)	121 (68.8)	55 (31.2)	*<0*.0001*
***Perceived risk of infection***				
same as other persons	592 (61.0)	481(81.2)	111(18.8)	
More than other persons	137 (14.1)	96 (70.1)	41 (29.9)	**0.002**
Less than other persons	242 (24.9)	205 (84.7)	37 (15.3)	

**Table 4:** Respect of hygiene rules and associated factors.

Variables	Respondent (N)	Respect Hygiene rules		p-value
		**YES**	**NO**	
**Gender**				
Male	422 (43.5)	387 (91.7)	**35 (8.3)**	*0*.017*
Female	549 (56.5)	**524 (95.5)**	**25 (4.5)**	
**Age (years)**				
5–14.	241 (24.8)	226 (93.8)	15 (6.2)	
15–29	325 (33.5)	304 (93.5)	21 (6.5)	
30–44	212 (21.8)	197 (92.9)	15 (7.1)	0.465
45–64	153 (15.8)	140 (91.5)	13 (8.5)	
≥65	40 (4.1)	40 (100.0)	0 (0.0)	
**Level of Education**				
No formal Education	57 (5.9)	54 (94.7)	3 (5.3)	
Primary	318 (32.7)	296 (93.1)	22 (6.9)	
Secondary	434 (44.7)	404 (93.1)	30 (6.9)	0.344
Tertiary	145 (14.9)	142 (97.9)	3 (2.1)	
Undergraduate/Postgraduate	17 (1.8)	15 (88.2)	2 (11.8)	
**Occupation**				
Housewife/maiden	74 (7.6)	71 (95.9)	3 (4.1)	
Student	402 (41.4)	382 (95.0)	20 (5.0)	
Salaried worker	54 (5.6)	51 (94.4)	3 (5.6)	
Informal worker	197 (20.3)	178 (90.4)	19 (9.6)	
Trading	116 (11.9)	107 (92.2)	9 (7.8)	0.282
Unemployed	68 (7.0)	65 (95.6)	3 (4.4)	
Retiree	32 (3.3)	32 (100.0)	0 (0.0)	
Farming	6 (0.6)	6 (100.0)	0 (0.0)	
Others	22 (2.3)	19 (86.4)	3 (13.6)	
**Symptoms**				
***COVID-19 compatible symptoms***				
YES	295 (30.4)	278 (94.2)	17 (5.8)	
NO	676 (69.6)	633 (93.6)	43 (6.4)	0.731
***COVID-19 symptoms***				
YES	115 (39.0)	106 (92.2)	9 (7.8)	
NO	180 (61.0)	172 (95.5)	8 (4.5)	0.229
***Symptom severity***				
Severe	45 (15.3)	42 (93.3)	3(6.7)	
Moderate	250 (84.7)	235 (94.0)	15 (6.0)	0.703
**Fear of contagion and perceived risk of infection**				
***Fear of viral contagion***				
YES	795 (81.9)	760 (95.6)	35 (4.4)	*<0*.0001*
NO	176 (18.1)	151 (85.8)	25 (14.2)	
***Perceived risk of infection***				
same as other persons	592 (61.0)	558 (94.3)	34 (5.7)	
More than other persons	137 (14.1)	122 (89.1)	15(10.9)	*0*.036*
Less than other persons	242 (24.9)	231 (95.5)	11 (4.5)	

**Table 5:** Sought testing and associated factors.

Variables	Respondent (N)	Sought testing		p-value
		**YES**	**NO**	
**Gender**				
Male	422 (43.5)	24 (5.7)	**398 (94.3)**	0.736
Female	549 (56.5)	**35 (56.4)**	**514 (93.6)**	
**Age (years)**				
5–14.	241 (24.8)	0 (0.0)	241 (100.0)	
15–29	325 (33.5)	24 (7.4)	301 (92.6)	
30–44	212 (21.8)	20 (9.4)	192 (90.6)	*<0*.0001*
45–64	153 (15.8)	11 (7.2)	142 (92.8)	
≥65	40 (4.1)	4 (10.0)	36 (90.0)	
**Level of Education**				
No formal Education	57 (5.9)	1(1.8)	56 (98.2)	
Primary	318 (32.7)	12(3.8)	306 (96.2)	
Secondary	434 (44.7)	18(4.1)	416 (95.9)	*<0*.0001*
Tertiary	145 (14.9)	22(15.2)	123 (84.8)	
Undergraduate/Postgraduate	17 (1.8)	6 (35.3)	11 (64.7)	
**Occupation**				
Housewife/maiden	74 (7.6)	4(5.4)	70(94.6)	
Student	402 (41.4)	15 (3.7)	387 (96.3)	
Salaried worker	54 (5.6)	10 (18.5)	44 (81.5)	
Informal worker	197 (20.3)	11 (5.6)	186 (94.4)	*0*.003*
Trading	116 (11.9)	9 (7.8)	107 (92.2)	
Unemployed	68 (7.0)	4 (5.9)	64 (94.1)	
Retiree	32 (3.3)	3 (9.4)	29 (90.6)	
Farming	6 (0.6)	0 (0.0)	6 (100.0)	
Others	22 (2.3)	3 (13.6)	19 (86.4)	
**Symptoms**				
***COVID-19 compatible symptoms***				
YES	295 (30.4)	29 (9.8)	266 (90.2)	
NO	676 (69.6)	30 (50.8)	646 (95.6)	*0*.001*
***COVID-19 symptoms***				
YES	115 (39.0)	14 (12.2)	101 (87.8)	
NO	180 (61.0)	15 (8.3)	165 (91.7)	0.287
***Symptom severity***				
Severe	45 (15.3)	4 (8.9)	41 (91.1)	
Moderate	250 (84.7)	25 (10.0)	225 (90.0)	0.852
**Fear of contagion and perceived risk of infection**				
***Fear of viral contagion***				
YES	795 (81.9)	44 (5.5)	751 (94.5)	
NO	176 (18.1)	15(8.5)	161(91.5)	0.222
***Perceived risk of infection***				
same as other persons	592 (61.0)	28 (4.7)	564 (95.3)	
More than other persons	137 (14.1)	17 (12.4)	120 (87.6)	*0*.003*
Less than other persons	242 (24.9)	14 (5.8)	228 (94.2)	

**Table 6:** Sought care and associated factors.

Variables	Respondent (N)	Sought care		p-value
		**YES**	**NO**	
**Gender**				
Male	422 (43.5)	33 (7.8)	**389 (92.2)**	0.761
Female	549 (56.5)	**40 (7.3)**	**509 (92.7)**	
**Age (years)**				
5–14.	241 (24.8)	12 (5.0)	229 (95.0)	
15–29	325 (33.5)	31 (9.5)	294 (90.5)	
30–44	212 (21.8)	16 (7.5)	196 (92.5)	0.185
45–64	153 (15.8)	8 (5.2)	145 (94.8)	
≥65	40 (4.1)	6 (15.0)	34 (85.0)	
**Level of Education**				
No formal Education	57 (5.9)	4 (7.0)	53 (93.0)	
Primary	318 (32.7)	20 (6.3)	298 (93.7)	
Secondary	434 (44.7)	32 (7.4)	402 (92.6)	0.117
Tertiary	145 (14.9)	13 (9.0)	132 (91.0)	
Undergraduate/Postgraduate	17 (1.8)	4 (23.5)	13 (76.5)	
**Occupation**				
Housewife/maiden	74 (7.6)	2(2.7)	72(97.3)	
Student	402 (41.4)	29 (7.2)	373 (92.8)	
Salaried worker	54 (5.6)	10 (18.5)	44 (81.5)	
Informal worker	197 (20.3)	12 (6.1)	185 (93.9)	
Trading	116 (11.9)	7 (6.0)	109 (94.0)	*0*.011*
Unemployed	68 (7.0)	8 (11.8)	60 (88.2)	
Retiree	32 (3.3)	5 (15.6)	27 (84.4)	
Farming	6 (0.6)	0 (0.0)	6 (100.0)	
Others	22 (2.3)	0 (0.0)	22 (100.0)	
**Symptoms**				
***COVID-19 compatible symptoms***				
YES	295 (30.4)	72 (24.4)	223 (75.6)	
NO	676 (69.6)	1 (0.1)	675 (99.9)	*<0*.0001*
***COVID-19 symptoms***				
YES	115 (39.0)	45 (39.1)	70 (60.9)	
NO	180 (61.0)	43 (23.9)	137 (76.1)	*0*.006*
***Symptom severity***				
Severe	45 (15.3)	12 (26.7)	33 (73.3)	
Moderate	250 (84.7)	76 (30.4)	174 (69.6)	0.676
**Fear of contagion and perceived risk of infection**				
***Fear of viral contagion***				
YES	795 (81.9)	59 (7.4)	736 (92.6)	
NO	176 (18.1)	14 (8.0)	162 (92.0)	0.812
***Perceived risk of infection***				
same as other persons	592 (61.0)	42 (7.1)	550 (92.9)	
More than other persons	137 (14.1)	12 (8.8)	125 (91.2)	0.778
Less than other persons	242 (24.9)	19 (8.3)	222 (91.7)	

Adherence to the lockdown was significantly associated with age; with the age group 45–64 being least compliant 24.8% (38/153), followed by the 30–44 age group, 18.9% (40/212), p<0.0001, (Table 2).

Adherence to SARS-CoV-2 lockdown was significantly associated with education. Those with a tertiary level of education (undergraduate and graduate) violated this rule most, 21.4% (31/145), followed by those with non-formal education, 19.3% (11/57), p = 0.026, (Table 2).

Profession was also significantly associated with adherence to SARS-CoV-2 lockdown; with traders breaking this rule more often, 32.8% (38/116), followed by salaried and informal workers, 29.6% (16/54) and 19.3% (38/178) respectively, p<0.0001. Similarly, the unemployed, 79.4% (54/68) and retirees, 68.8% (22/32), represented the groups with high proportion of individuals who did not stop working in violation of protection protocol, p<0.0001 (Table 2).

### SARS-CoV-2 preventive and social-behavioural practices according to fear of viral contagion and perceived risk of infection

A majority, 95.6% (760/795) of those who feared getting infected respected hygiene rules (p<0.0001) (Table 4). Among these individuals, up to 49.3% (392/795) and 16.9% (134/795) did not stop work and did not respect social distancing rules respectively, (p = 0.026 and p<0.0001) (Tables 3 and 7). In addition, up to 69.8% (555/795) felt healthcare facilities were dangerous for getting infected, p = 0.002, (Table 8).

**Table 7:** Stopped work and associated factors.

Variables	Respondent (N)	Stopped work		p-value
		**YES**	**NO**	
**Gender**				
Male	422 (43.5)	221 (52.4)	**201 (47.6)**	0.071
Female	549 (56.5)	**256 (46.6)**	**293 (53.4)**	
**Age (years)**				
5–14.	241 (24.8)	117 (48.5)	124 (51.5)	
15–29	325 (33.5)	171 (52.6)	154 (47.4)	
30–44	212 (21.8)	110 (51.9)	102 (48.1)	0.055
45–64	153 (15.8)	66 (43.1)	87 (56.9)	
≥65	40 (4.1)	14 (35.0)	26 (65.0)	
**Level of Education**				
No formal Education	57 (5.9)	22 (38.6)	35 (61.4)	
Primary	318 (32.7)	158 (49.7)	160 (50.3)	
Secondary	434 (44.7)	206 (47.5)	228 (52.5)	0.056
Tertiary	145 (14.9)	78 (53.8)	67 (46.2)	
Undergraduate/Postgraduate	17 (1.8)	13 (76.5)	4 (23.5)	
**Occupation**				
Housewife/maiden	74 (7.6)	31 (41.9)	43 (58.1)	
Student	402 (41.4)	214(53.2)	188(46.8)	
Salaried worker	54 (5.6)	25(46.3)	29(53.7)	
Informal worker	197 (20.3)	111 (56.3)	86 (43.7)	<0.0001*
Trading	116 (11.9)	62 (53.4)	54 (46.6)	
Unemployed	68 (7.0)	14(20.6)	54(79.4)	
Retiree	32 (3.3)	10 (31.2)	22 (68.8)	
Farming	6 (0.6)	2 (33.3)	4 (66.7)	
Others	22 (2.3)	8 (36.4)	14 (63.6)	
**Symptoms**				
***COVID-19 compatible symptoms***				
YES	295 (30.4)	152 (51.5)	143 (48.5)	
NO	676 (69.6)	325 (48.1)	351 (51.9)	0.281
***COVID-19 symptoms***				
YES	115 (39.0)	68 (59.1)	47(40.9)	
NO	180 (61.0)	84 (46.7)	96 (53.3)	*0*.041*
***Symptom severity***				
Severe	45 (15.3)	24 (53.3)	21 (46.7)	
Moderate	250 (84.7)	128 (51.2)	122 (48.8)	0.682
**Fear of contagion and perceived risk of infection**				
***Fear of viral contagion***				
YES	795 (81.9)	403 (50.7)	392 (49.3)	
NO	176 (18.1)	74 (42.0)	102 (58.0)	0.222
***Perceived risk of infection***				
same as other persons	592 (61.0)	282 (47.6)	310 (52.4)	
More than other persons	137 (14.1)	65 (47.4)	72 (52.6)	*0*.003*
Less than other persons	242 (24.9)	130 (53.7)	112 (46.3)	

**Table 8:** Fear of healthcare centers and associated factors.

Variables	Respondent (N)	Think health centres are dangerous sites for infection		p-value
		**YES**	**NO**	
**Gender**				
Male	422 (43.5)	284 (67.3)	**138 (32.7)**	0.520
Female	549 (56.5)	**359 (65.4)**	**190 (34.6)**	
**Age (years)**				
5–14.	241 (24.8)	174 (72.2)	67 (27.8)	
15–29	325 (33.5)	221 (68.0)	104 (32.0)	
30–44	212 (21.8)	137(64.6)	75 (35.4)	*0*.008*
45–64	153 (15.8)	91 (59.5)	62 (40.5)	
≥65	40 (4.1)	21 (52.5)	19 (47.5)	
**Level of Education**				
No formal Education	57 (5.9)	39 (68.4)	18 (31.6)	
Primary	318 (32.7)	216 (67.9)	102 (32.1)	
Secondary	434 (44.7)	280 (64.5)	154 (35.5)	*<0*.0001*
Tertiary	145 (14.9)	97 (66.9)	48 (33.1)	
Undergraduate/Postgraduate	17 (1.8)	11 (64.7)	6 (35.3)	
**Occupation**				
Housewife/maiden	74 (7.6)	46 (62.2)	28 (37.8)	
Student	402 (41.4)	284 (70.6)	118 (29.4)	
Salaried worker	54 (5.6)	34 (63.0)	20 (37.0)	
Informal worker	197 (20.3)	122 (61.9)	75 (38.1)	0.094
Trading	116 (11.9)	79 (68.1)	37 (31.9)	
Unemployed	68 (7.0)	45 (66.2)	23 (33.8)	
Retiree	32 (3.3)	14 (43.8)	18 (56.2)	
Farming	6 (0.6)	5 (83.3)	1 (16.7)	
Others	22 (2.3)	14 (63.6)	8 (36.4)	
**Symptoms**				
***COVID-19 compatible symptoms***				
YES	295 (30.4)	193 (65.4)	102 (34.6)	
NO	676 (69.6)	450 (66.6)	226 (33.4)	0.703
***COVID-19 symptoms***				
YES	115 (39.0)	72 (62.6)	43 (37.4)	
NO	180 (61.0)	120 (66.7)	60 (33.3)	0.436
***Symptom severity***				
Severe	45 (15.3)	25 (55.6)	20 (44.4)	
Moderate	250 (84.7)	167(66.8)	83 (33.2)	0.200
**Fear of contagion and perceived risk of infection**				
***Fear of viral contagion***				
YES	795 (81.9)	555 (69.8)	240 (30.2)	
NO	176 (18.1)	88 (50.0)	88 (50.0)	0.394
***Perceived risk of infection***				
same as other persons	592 (61.0)	410 (69.3)	182 (30.7)	
More than other persons	137 (14.1)	89 (65.0)	48 (35.0)	0.022*
Less than other persons	242 (24.9)	144 (59.5)	98 (40.5)	

Those who felt they have a higher risk of getting infected than other persons violated the lockdown rule more often, 27.7% (38/137, p<0.0001) (Table 2), as well as social distancing (Table 3), 29.9% (41/137) and hygiene rules10.9% (15/137) (Table 4), in this order (p = 0.002 and p = 0.036, respectively).

### SARS-CoV-2 preventive and social-behavioural practices according to symptoms

Among the 295 participants with COVID-19 compatible symptoms, 39.0% (115/295) presented symptoms of COVID-19. Of note, up to 75.6% (223/295) did not seek medical consultation (Table 6), while only 9.8% (29/295) of these symptomatic individuals reportedly took an antigen test (Table 5). Similarly, among those who did not stop working in violation of barrier measures (respect of hygiene, social distancing, and lockdown rules), 40.9% (47/115) reported having had symptoms of SARS-CoV-2 infection, p = 0.041 (Table 7).

In all, 15.3% (45/295) of the symptomatic respondents reported having experienced severe clinical manifestations, while 84.7% (250/295) experienced moderate manifestations. Of these 45 respondents, 73.3% (33/45) reportedly did not seek medical consultation (Table 6), while only 8.9 (4/45) were tested using a SARS CoV-2 antigen test (Table 5).

### SARS-CoV-2 preventive and social-behavioural practices according to SARS-CoV-2 IgG positivity

More men were detected positive for SARS-CoV-2 IgG than women; 36.5% (154/422) in males versus 27.0% (148/549) in females, p = 0.021. The age group ≥ 65 years recorded the highest prevalence of SARS-CoV-2 IgG positivity, 37.5% (15/40), followed by those 45–64 years, 33.3% (51/153), p = 0.080. Conversely, those aged 5–14 years recorded the least proportion of IgG cases, 28.6% (69/241), p = 0.080 (Table 9). Postgraduates had a higher proportion of IgG cases, 35.3% (6/17), followed by undergraduates and graduates, 33.8% (49/145) while the least was observed among those with non-formal education, 21.1% (12/57), p = 0.369. Housewives/maidens and informal workers reported the highest proportion of SARS-CoV-2 IgG cases (43.2% (32/74) and 35.5% (70/197), respectively) (Table 9).

**Table 9:** SARS-CoV-2 IgG and associated factors.

Variables	Respondent (N)	SARS-CoV-2 IgG		p-value
		**YES**	**NO**	
**Gender**				
Male	422 (43.5)	**154 (36.5)**	**268 (63.5)**	*0*.021*
Female	549 (56.5)	148(27.0)	401(73.0)	
**Age (years)**				
5–14.	241 (24.8)	69 (28.6)	172 (71.4)	
15–29	325 (33.5)	98 (30.2)	227 (69.8)	
30–44	212 (21.8)	69 (32.5)	143(67.5)	0.080
45–64	153 (15.8)	51 (33.3)	102 (66.7)	
≥65	40 (4.1)	15 (37.5)	25 (62.5)	
**Level of Education**				
No formal Education	57 (5.9)	12 (21.1)	45 (78.9)	
Primary	318 (32.7)	106 (33.3)	212 (66.7)	
Secondary	434 (44.7)	129 (29.7)	305 (70.3)	0.369
Tertiary	145 (14.9)	49 (33.8)	96 (66.2)	
Undergraduate/Postgraduate	17 (1.8)	6 (35.3)	11 (64.7)	
**Occupation**				
Housewife/maiden	74 (7.6)	32 (43.2)	42 (56.8)	
Student	402 (41.4)	121 (30.1)	281 (69.9)	
Salaried worker	54 (5.6)	12 (22.2)	42 (77.8)	
Informal worker	197 (20.3)	70 (35.5)	127 (64.5)	0.992
Trading	116 (11.9)	30 (25.9)	86 (74.1)	
Unemployed	68 (7.0)	22 (32.4)	46 (67.6)	
Retiree	32 (3.3)	8 (25.0)	24 (75.0)	
Farming	6 (0.6)	1 (16.7)	5 (83.3)	
Others	22 (2.3)	6 (27.3)	16 (72.7)	
**Symptoms**				
***COVID-19 compatible symptoms***				
YES	295 (30.4)	108 (36.6)	187(63.4)	
NO	676 (69.6)	194 (28.7)	482(71.3)	*0*.014*
***COVID-19 symptoms***				
YES	115 (39.0)	50 (43.5)	65 (56.5)	
NO	180 (61.0)	58 (32.2)	122 (67.8)	0.050
***Symptom severity***				
Severe	45 (15.3)	21 (51.1)	22 (48.9)	
Moderate	250 (84.7)	83 (32.4)	169 (67.6)	*0*.044*
**Fear of contagion and perceived risk of infection**				
***Fear of viral contagion***				
YES	795 (81.9)	252 (31.7)	543(68.3)	
NO	176 (18.1)	50 (28.4)	126 (71.6)	0.394
***Perceived risk of infection***				
same as other persons	592 (61.0)	175 (29.6)	417(70.4)	
More than other persons	137 (14.1)	58 (42.3)	79 (57.7)	*0*.009*
Less than other persons	242 (24.9)	69 (28.5)	173 (71.5)	

Of 795 respondents who feared catching the virus, 31.7% (252/795) were positive for SARS-CoV-2 IgG. Regarding the perceived risk of contracting the disease, those who felt they were more likely to contract the virus reported a higher proportion of IgG cases, 42.3% (58/137), (Table 9).

**Fig 2:**
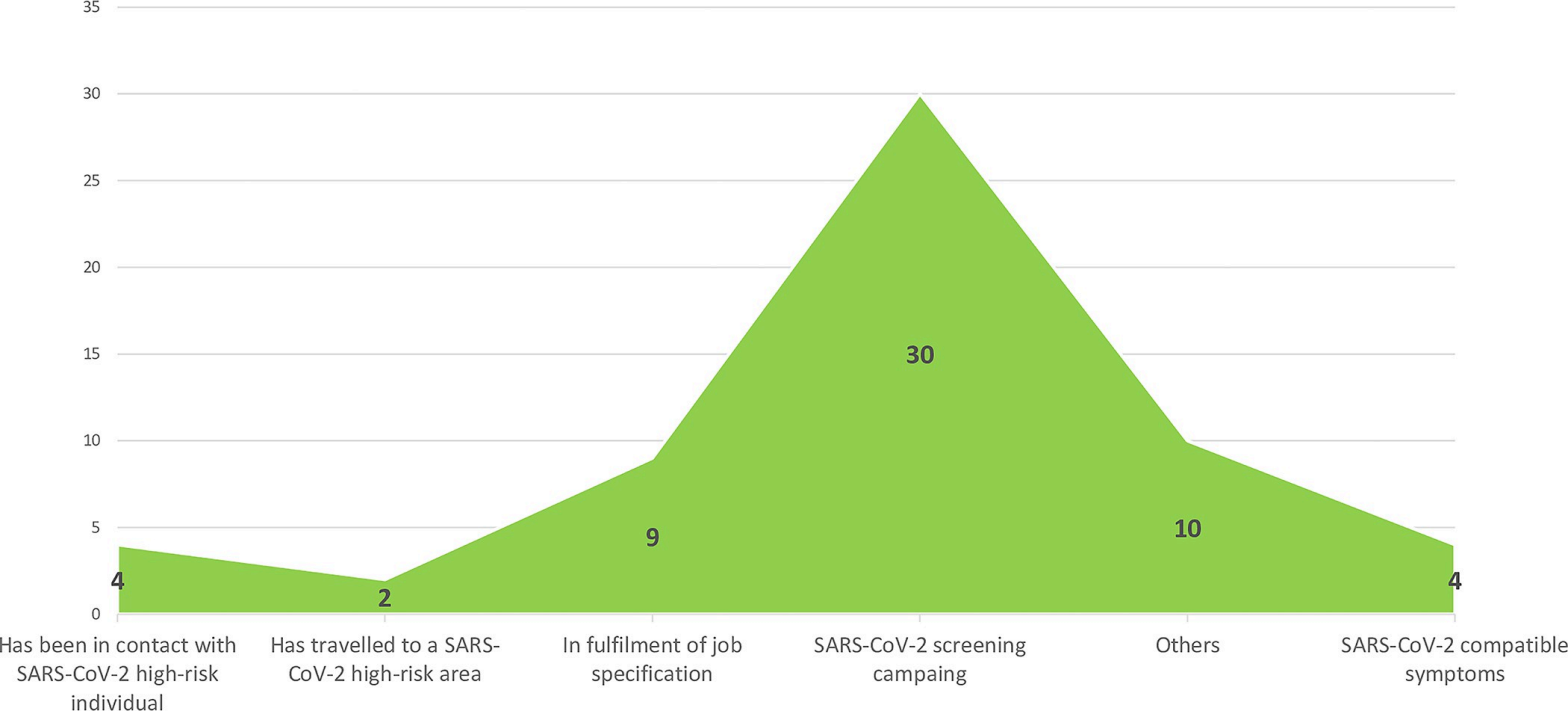
Respondents’ SARS CoV-2 Ag testing motivation.

Of the 295 symptomatic respondents observed, 36.6% (108/295) had SARS-CoV-2 IgG, p = 0.014 while 43.5% (50/115) respondents with COVID-19 symptoms tested positive for SARS-CoV-2 IgG, (Table 9). Only 16.9% (10/59) of those who did a SARS-CoV-2 test did so for healthcare and/or risk-related reasons [60% (6/10) of whom were reactive for SARS-CoV-2-IgG]. The rest, 83.1% (49/59) did take the antigen test for reasons not related to healthcare and/or risk of exposure [40.8% (20/49) of whom were reactive for SARS-CoV-2-IgG], (Fig 2).

### Predicted factors associated to social behavioural practices and SARS-CoV-2 IgG

After adjusting for gender, age, level of education, and occupation to COVID-19 preventive social-behavioural practices, binary logistic regression analysis showed that females were significantly associated with ’’respect of hygiene rules’’, ’’respect of social distancing rules’’, and ’’obey lockdown rule’’, (p = 0.019, OR: 1.98; p = 0.016, OR: 1.52; and p = 0.005, OR: 1.76, respectively), and male with the ’’SARS-CoV-2 IgG’’ positivity (p = 0.013, OR:0.69). Age was significantly associated to ’’obey lockdown rule’’ (15–29 years), ’’sought care’’ (≥ 65 years) and ’’think health centres are sites dangerous for infection” (≥ 65 years) (p = 0.035, OR: 0.43; p = 0.003, OR: 5.03 and p = 0.031, OR: 0.39, respectively). Occupation was associated with ’’obey lockdown rule’’ (salaried worker p = 0.023, OR: 0.29, and trading p = 0.001, OR: 0.23), ’’stopped work’’ (unemployed p = 0.003, OR: 0.32), and ’’sought care’’ (salaried worker p = 0.044, OR: 2.74) (Table 10).

**Table 10:** Predicted factors associated to social behavioural practices and SARS-CoV-2 IgG.

Variables	**Respect hygiene rules**		OR: 95% CI	p-value
**Gender**	**YES**	**NO**		
Male	387 (91.7)	35 (8.3)	1	
Female	524 (95.5)	25 (4.5)	1.98 (1.12–3.52)	*0*.019*
Variables	**Respect social distancing rules**		OR: 95% CI	p-value
**Gender**	**YES**	**NO**		
Male	325 (77.0)	97 (23.0)	1	
Female	457 (83.2)	92 (16.8)	1.52 (1.02–2.14)	*0*.016*
Variables	**Obey lockdown rule**		OR: 95% CI	p-value
**Gender**	**YES**	**NO**		
Male	342 (81.0)	80 (19.0)	1	
Female	481 (87.6)	68 (12.4)	1.76 (1.19–2.60)	*0*.005*
**Age (years)**	**YES**	**NO**		
5–14.	226 (93.8)	15 (6.2)	1	
15–29	304 (93.5)	21 (6.5)	0.43 (0.20–0.94)	*0*.035*
30–44	197(92.9)	15(7.1)	0.56 (0.23–1.38)	0.210
45–64	140 (91.5)	13 (8.5)	0.55 (0.22–1.34)	0.196
≥ 65	40 (100.0)	0 (0.0)	0.71 (0.18–2.74)	0.619
**Occupation**	**YES**	**NO**		
Housewife/maiden	68 (91.9)	6 (8.1)	1	
Student	367 (91.3)	35 (8.7)	1.03 (0.37–2.89)	0.946
Salaried worker	38 (70.4)	16 (29.6)	0.29 (0.10–0.84)	*0*.023*
Informal worker	159 (80.7)	38 (19.3)	0.55 (0.21–1.44)	0.223
Trading	78 (67.2)	38 (32.8)	0.23 (0.10–0.56)	*0*.001*
Unemployed	60 (88.2)	8 (11.8)	0.83 (0.27–2.57)	0.742
Retiree	28 (87.5)	4 (12.5)	0.71 (0.16–3.18)	0.651
Farming	6 (100.0)	0 (0.0)	/	0.999
Others	19 (86.4)	3 (13.6)	0.56 (0.12–2.57)	0.458
Variables	**Stopped work**		OR: 95% CI	p-value
**Occupation**	**YES**	**NO**		
Housewife/maiden	31 (41.9)	43 (58.1)	1	
Student	214(53.2)	188(46.8)	1.69 (0.90–3.18)	0.105
Salaried worker	25(46.3)	29(53.7)	0.89 (0.41–1.92)	0.766
Informal worker	111 (56.3)	86 (43.7)	1.58 (0.89–2.81)	0.119
Trading	62 (53.4)	54 (46.6)	1.52 (0.83–2.80)	0.174
Unemployed	14(20.6)	54(79.4)	0.32 (0.15–0.68)	*0*.003*
Retiree	10 (31.2)	22 (68.8)	0.75 (0.27–2.05)	0.568
Farming	2 (33.3)	4 (66.7)	0.68 (0.11–4.05)	0.671
Others	8 (36.4)	14 (63.6)	0.92 (0.34–2.55)	0.879
Variables	**Sought care**		OR: 95% CI	p-value
**Age (years)**	**YES**	**NO**		
5–14.	12 (5.0)	229 (95.0)	1	
15–29	31 (9.5)	294 (90.5)	1.81 (0.90–3.67)	0.098
30–44	16 (7.5)	196 (92.5)	1.72 (0.73–4.10)	0.217
45–64	8 (5.2)	145 (94.8)	2.23 (0.91–4.56)	0.080
≥ 65	6 (15.0)	34 (85.0)	5.03 (1.72–14.78)	*0*.003*
**Occupation**	**YES**	**NO**		
Housewife/maiden	2(2.7)	72(97.3)	1	
Student	29 (7.2)	373 (92.8)	1.44 (0.57–3.62)	0.439
Salaried worker	10 (18.5)	44 (81.5)	2.74 (1.02–7.30)	*0*.044*
Informal worker	12 (6.1)	185 (93.9)	1.35 (0.57–3.17)	0.497
Trading	7 (6.0)	109 (94.0)	1.28 (0.52–3.17)	0.588
Unemployed	8 (11.8)	60 (88.2)	2.14 (0.85–5.42)	0.107
Retiree	5 (15.6)	27 (84.4)	1.55 (0.47–5.10)	0.473
Farming	0 (0.0)	6 (100.0)	1.33 (0.13–13.43)	0.807
Others	0 (0.0)	22 (100.0)	0.87 (0.17–4.53)	0.868
Variables	**Think health centres are sites dangerous for infection**		OR: 95% CI	p-value
**Age (years)**	**YES**	**NO**		
5–14.	174 (72.2)	67 (27.8)	1	
15–29	221 (68.0)	104 (32.0)	0.83 (0.51–1.35)	0.454
30–44	137(64.6)	75 (35.4)	0.71 (0.39–1.31)	0.274
45–64	91 (59.5)	62 (40.5)	0.61 (0.32–1.16)	0.134
≥ 65	21 (52.5)	19 (47.5)	0.39 (0.17–0.92)	*0*.031*
Variables	**SARS-CoV-2 IgG**		OR: 95% CI	p-value
**Gender**	**YES**	**NO**		
Male	154 (36.5)	268 (63.5)	1	
Female	148(27.0)	401(73.0)	0.69 (0.51–0.92)	*0*.013*
**Age (years)**				
5–14.	69 (28.6)	172 (71.4)	1	
15–29	98 (30.2)	227 (69.8)	1.20 (0.73–1.97)	0.47
30–44	69 (32.5)	143(67.5)	1.37 (0.73–2.56)	0.32
45–64	51 (33.3)	102 (66.7)	1.42 (0.73–2.74)	0.30
≥ 65	15 (37.5)	25 (62.5)	2.72 (1.11–6.70)	*0*.020*

## Discussion

We aimed to assess SARS-CoV-2 preventive, social-behavioural practices, and SARS-CoV-2 IgG among residents in the city of Yaounde. Many residents within Cite Verte health district zone highly exposed to COVID-19, perceived healthcare facilities as high-risk zones for SARS-CoV-2 infection and consequently did not seek medical interventions. The 31% SARS-CoV-2 IgG positivity obtained is a reflection of the degree of viral transmission which is way above officially reported statistics at the time. This information may enable tailored interventions for specific social and behavioural practices, which may help in controlling any future pandemic in our context.

As of the time this study was conducted, the most effective recommended mitigating transmission measures were lockdowns, social distancing, hygiene rules, and isolation [18]. We observed that up to 93.8% of the respondents respected basic hygiene rules while 84.8% and 80.5% complied with lockdown and social distancing rules, respectively. A survey conducted in some parts of Africa between March and May 2020 [14–16] documented very good adherence to preventive measures, which increased steadily during the exponential phase of the outbreak (March to August 2020). Given the devastating reports of COVID-19 morbidity and mortality outside of SSA (with the exception of South Africa), the positive national response coupled with fear (as it is the case with any new disease outbreak) led to increase awareness and compliance with these barrier measures [19]. This partly explains why most of the participants enrolled in this study like in similar studies in some parts of Africa reported satisfactory adherence to preventive measures. The reported SARS-CoV-2 IgG prevalence of 31.1%, suggests a higher community transmission of the virus than the country’s 22692 cases reported officially in November 2020 [20]; thus, depicting loopholes in acclaimed adherence to preventive measures. This is a scenario that could be replicated in the face of any disease outbreak. A study conducted in West Africa noted a high level of asymptomatic viral infection among participants, which was the most likely cause of community transmission [21], witch our study concords.

Based on socio-demographic characteristics, females respected barriers measures of hygiene, social distancing, and confinement more than their male counterparts. Correspondingly, SARS-CoV-2 IgG test showed a slightly lower positivity rate (27.0% against 36.5%) in favour of females. According to the Central Bureau of Census and Population Studies (BUCREP) June report, women respect recommended barrier measures of regular wearing of face masks, regular hand washing, and restriction of unnecessary outings more (76.4% against 62.0%) [22].

Men are generally bread winners who have as responsibility to furnish the daily needs of their households. To achieve this, many were compelled to defy restriction rules. Furthermore, to many, Covid-19 disease is a “white man’s thing” with rules in opposition to cultural norms [18], people often saw no reason to fully respect barrier measures. This is a phenomenon that can be encountered each time a new disease outbreak occurs.

Age-related behaviours played an important role in the response to the pandemic. The elderly, for example, were identified as a high-risk group for SARS-CoV-2 infection [23] and to take extra precautions to protect themselves. This explains why those ≥ 65 years old respected barrier measures more than those within the active age groups of 15–29 and 30–44. People ≥ 65 years are undoubtedly more vulnerable to SARS-CoV-2 infection, consequently, were more diligent in following recommendations than those within the active age class who often perceive preventive measures as anti-social [18]. The perception of invincibility or a lack of understanding of the potential consequences of actions among young adults led to lapses in adhering to preventive measures. Despite the high level of adherence to preventive rules, the elderely, however, had a higher proportion of SARS-CoV-2 IgG positive individuals (37.5%) highlighting the need to tailor preventive interventions.

For future health communication strategies, it will be important to tailor messages to different age groups. For older adults, it may be effective to stress the importance of following guidelines to protect their health. For young adults, messages that appeal to their sense of responsibility to protect others may be more effective.

There’s usually disparity in perception and awareness in societies regarding some diseases or at least each time a new disease outbreak ensues. This is reflected here in the degree of disparity in adherence to preventive measures based on educational level. Respondents with undergraduate and graduate diplomas ignored lockdown rules more (21.4%) while those who breached hygiene and social distancing rules more were those with postgraduate (11.8%) and secondary (21.2%) education, respectively. Controversies about the virus within our context perhaps left many perplexed. Discriminating misinformation during the intense infodemic period was quite challenging in our context [24]. This led to discordance between the respect of protective measures and SARS-CoV-2 IgG finding among participants [24].

Participants with a primary education had the least health-seeking attitude, (6.3% for medical consultation) and the highest proportion of individuals who felt healthcare facilities were dangerous for getting infected, (67.9%). SARS-CoV-2 IgG result showed that those with undergraduate and graduate diplomas had the highest proportion of cases, 33.8% (49/145). Housewives and maidens were more exposed to SARS-CoV-2 and reported the highest proportion of SARS-CoV-2 IgG cases, 43.2% (32/74). Amid lockdown, only the sales of foodstuffs and some essential services were allowed and the buying of these commodities were largely being carried out by housewives and maidens who were constantly being exposed to the virus. This result seems paradox, giving that housewives and maidens did not necessarily ignore lockdown measures (as compared to traders for example). Thus, highlighting some challenges that were faced during the first three waves of the pandemic and the need for tailored Risk Communication and Community Engagement (RCCE) strategies targeting the implementation of barrier measures.

SARS-CoV-2 infection perception and perceived vulnerability were significantly associated with regular adherence to hygiene, social distancing, and lockdown rules. This survey showed that the more fearful people were about the viral contagion, the more they obeyed barrier measures. Surprisingly, those who felt they were more likely to get the virus breached barrier measures more and as a result SARS-CoV-2-IgG prevalence was higher (42.3%). A study investigating risk perception among residents in seven SSA countries noted up to 33% perceived risk of getting the virus [25] whereas here, the self-estimated prevalence was 14.1%.Unlike the study conducted in the seven SSA countries that were nationwide, this study was limited to a single health district. Thus, justifying the SARS-CoV-2 perceived risk difference between these two studies.

Despite the symptoms observed (30.4% Flu symptoms and 30.4% SARS-CoV-2 suspected cases), a majority did not seek medical intervention, with only 24.4% and 9.8% reportedly sought medical consultation and SARS-CoV-2 –Ag screening in this order. Furthermore, a scenario as observed here where many, (66.2%) felt healthcare facilities as potential sites for SARS-CoV-2 contagion somehow suggests why many failed to seek medical intervention; with up to 83.1% of those who did the test did so for reasons not related to healthcare and/or risk ofexposure. Hence, a potential social behavioural risk indicator that perhaps propelled the disease dissemination; undermining the effective implementation of the COVID-19 3T strategy of “Test, Trace and Treat”.

## Conclusion

Even though the level of adherence to SARS-CoV-2 preventive measures was acceptable, SARS-CoV-2 IgG profile proved the contrary. Many perceived healthcare facilities as high-risk zones for SARS-CoV-2 infection and consequently did not seek medical interventions. Thus, in the context of such a pandemic, advocacy on risk communication and community engagement for health-seeking attitudes, interventions should have a special emphasis on groups who are more prone to not adhering to public health guidance which in this case is men. This can partly be achieved through using relatable language, highlighting potential consequences of not following guidelines, providing clear guidance, and utilizing trusted messengers such as community leaders/mobilisation agents or healthcare providers. Future research should consider studying the impact of fear of healthcare facilities on health-seeking behaviour during pandemics.
